# Prevalence of Clinical Anxiety, Clinical Depression and Associated Risk Factors in Chinese Young and Middle-Aged Patients with Osteonecrosis of the Femoral Head

**DOI:** 10.1371/journal.pone.0120234

**Published:** 2015-03-19

**Authors:** Sheng-Bao Chen, Hai Hu, You-Shui Gao, Hai-Yan He, Dong-Xu Jin, Chang-Qing Zhang

**Affiliations:** Department of Orthopedic Surgery, Shanghai Jiao Tong University Affiliated Sixth People’s Hospital, Shanghai, China; National Center of Neurology and Psychiatry, JAPAN

## Abstract

**Objective:**

To investigate the prevalence of clinical anxiety and clinical depression in Chinese young and mid-aged patients with osteonecrosis of the femoral head (ONFH) and to analyze their potential risk factors.

**Methods:**

Two hundred and sixteen Chinese patients with ONFH were consecutively enrolled in this cross-sectional study from January 2010 to December 2010. The Zung self-rating anxiety scale (SAS) and the Zung self-rating depression scale (SDS) were used to assess the prevalence of clinical anxiety and clinical depression. An additional questionnaire containing seventeen items of potential risk factors was completed by all patients. Binary logistic regression analysis was employed to reveal potential risk factors of anxiety and depression.

**Results:**

The prevalence of clinical anxiety and clinical depression was 20.4% and 21.8% in Chinese young and middle-aged patients with ONFH, respectively. Binary regression analysis showed that independent risk factors correlated with high incidence of clinical anxiety included involved femoral head (OR = 3.168, 95% *CI*: 1.496 - 6.708) and stages of ONFH (OR_IV-V / II_ = 5.383, 95% *CI*: 1.664-17.416). Independent risk factors correlated with high incidence of depression included gender (OR = 2.853, 95% *CI*: 1.467-5.778), comorbid diseases (OR = 4.243, 95% *CI*: 1.940-9.278) and stages of the disease (OR _IV-V/II_ = 16.963, 95% *CI*: 4.404-65.331).

**Conclusions:**

Patients with bilateral ONFH are inclined to have clinical anxiety, while female patients and patients with comorbid diseases might tend to get clinical depression. Advanced stages of ONFH are independent risk factors for both clinical anxiety and clinical depression.

## Introduction

Osteonecrosis of the femoral head (ONFH) is a refractory disease that can lead to collapse of the articular cartilage and disabling osteoarthritis of the hip. Despite of those asymptomatic ONFH, there is a high prevalence (59%) of cases who progressed to symptomatic phrases and whose femoral heads collapsed without treatment [1]. In addition, nearly 12% of total hip arthroplasties were performed to manage advanced ONFH per year in the United States [2]. Epidemiologic studies showed an average prevalence was 28.91 per 100000 populations in Korea, and nearly 20,000 new cases were diagnosed annually in the United States [3–4], ONFH mainly affects young and middle-aged people at their ages of 20–50 years, characterized by nonspecific symptoms of groin pain and restricted motion of the hip. The persisting pain and movement disorder would reduce or deprive of living and working ability, and finally cause psychological problems in patients with ONFH [5–6].

In the past decade, the importance of mental status and potential risk factors in patients with musculoskeletal disorders has been noted [7–9]. For ONFH, the majority of previous clinical reports just focused on the methods of diagnosis and treatment [10–11]. However, no studies reported the mental status in patients with ONFH, particularly their clinical anxiety and clinical depression. Therefore, we made a cross-sectional epidemiological investigation to reveal the prevalence of clinical anxiety and clinical depression in Chinese young and middle-aged patients with ONFH. Moreover, the potential risk factors correlated with anxiety and depression were investigated thoroughly.

### Patients and Methods

This cross-sectional investigation enrolled patients who were diagnosed as ONFH and hospitalized for operations in Shanghai Sixth People Hospital between January 2010 and December 2010. The age of the enrolled patients ranged from 14 to 60 years old. The definitive diagnosis of ONFH was comprehensively based on clinical symptoms, radiographic findings and characteristic disease history (such as steroid medication and alcohol abuse). Zung self-rating anxiety scale (SAS) [12] and self-rating depression scale (SDS) [13] were employed to investigate clinical anxiety and clinical depression respectively. Additionally, potential risk factors correlated with the psychological status were noted for further analysis. All investigations were performed before operation. Written consent has been obtained from all participants or from their parents, next of kin, caretakers, or guardians in the participants younger than 18 years old. The study protocol was approved by the Medical Ethics Committee of Shanghai Sixth People’s Hospital (201020), and the research was carried out in compliance with the Helsinki Declaration.

### Questionnaires

SAS and SDS were employed. As valid tools, these two scales have been widely used to evaluate clinical anxiety and clinical depression [7,14–17]. The reliability and validity of the Chinese version of SAS and SDS scales have been confirmed in previous epidemiological surveys [15–17]. Both of them included 20 questions, the cutoff point of SAS score ≥ 50 and SDS score ≥ 53 considered as the presence of clinical anxiety and clinical depression in the Chinese populations, respectively.

An additional questionnaire was used to record all factors possibly associated with clinical anxiety and clinical depression. The design of questionnaire was finalized after repeatedly discussion and pilot surveyed, the questionnaire included general demographic information on 17 items (Table 1). These factors were listed as follow: age (Year), gender (Male/Female), education (Primary school/Middle school/High school/College), occupation (Physical labor/Farmer/White Collar/Student/Retailer/Self-employed), marriage (Married/Single/Divorced/Widowed), employment status (Employed/On leave/Unemployed/Retired) and salary per month (< ¥2000/¥2000 ~ ¥5000/> ¥5000), smoking (Never/Quitted/Smoking) and drinking (Never/Quitted/Occasionally/Alcoholic), comorbid diseases (No/Yes), etiology of necrosis (Traumatic/Steroid-induced/Alcohol-induced/Idiopathic), period of the disease, involved femoral head (Unilateral/Bilateral), Steinberg stage (II/III/IV and V), pain scores on the visual analog scale (VAS) (0–10cm), knowledge of ONFH (None/Limited/Clear) and Harris hip score (HHS).

**Table 1:** The baseline characteristic distribution of 216 Chinese patients with ONFH.

Items	N (%)
**Gender**	
Male	162 (75.0%)
Female	54 (25.0%)
**Age** (x̄ ± s, years)	35.2 ± 10.6 (15–60)
**Education**	
Primary school	33 (15.3%)
Middle school	89 (41.2%)
High school	64 (29.6%)
College	30 (13.9%)
**Marriage**	
Single	65 (30.1%)
Married	148 (68.5%)
Divorced/Widowed	3 (1.4%)
**Occupation**	
Physical labor	80 (37.0%)
Farmer	31 (14.4%)
White collar	32 (14.8%)
Student	22 (10.2%)
Retailer	11 (5.1%)
Self employed	40 (18.5%)
**Employment status**	
Employed	48 (22.2%)
On leave	39 (18.1%)
Unemployed	125 (57.9%)
Retired	4 (1.9%)
**Salary per month**	
< ¥2000	104 (48.2%)
¥2000~5000	88 (40.7%)
> ¥5000	24 (11.1%)
**Smoking**	
Never	137 (63.4%)
Quitted	22 (10.2%)
Smoking	57 (26.4%)
**Alcohol**	
Never	123 (56.9%)
Quitted	19 (8.8%)
Occasionally	59 (27.3%)
Alcoholic	15 (6.9%)
**Disease period** (months)	
Median (25% -75% quantiles)	1.7 (0.2–5.0)
**Etiology**	
Traumatic	52 (24.1%)
Steroid-induced	70 (32.4%)
Alcoholic-induced	45 (20.8%)
Idiopathic	49 (22.7%)
**Steinberg Stage**	
II	49 (22.7%)
III	108 (50.0%)
IV and V	59 (27.3%)
**Comorbid diseases**	
No	136 (63.0%)
Yes	80 (37.0%)
**Involved femoral head**	
Unilateral	112 (51.9%)
Bilateral	104 (48.2%)
**Knowledge of ONFH**	
None	57 (26.4%)
Limited	140 (64.8%)
Clear	19 (8.8%)
**Pain score on (VAS)**	
(x̄ ± s, range)	4.8 ± 1.8 (1–10)
**Harris hip score (HHS)**	
(x̄ ± s, range)	71.0 ± 13.2 (20–96)

The Steinberg stage is one of classification of ONFH based on radiology, which consists of stage I to V and marching its progression process from the pre-symptomatic phase to end-stage osteoarthritis [18]. The Harris hip score is an assessment scale widely used to assess hip functional status in the field of orthopedic surgery, the cumulative scores consist of nine parts: pain, the usage of supportive devices, walking distance, presence of a limp, activities (the use of shoes and socks), stair climbing ability, ability to use public transportation, ability to sit, and factors regarding range of motion. A poor functional status is considered a total score below 70; fair 70–90; good 80–90; and excellent 90–100. Besides self-rating of SAS and SDS completed by themselves, the additional questionnaire has been filled in the form of interview by the professional investigators.

### Data analysis

All questionnaires were recorded in Epidata 3.0 (EpiData Association, Odense, Denmark), and checked by two independent researchers. Then the data were transferred to SPSS 14.0 (SPSS Inc. Chicago, IL, USA) for statistical analysis. There were three kinds of data: quantitative data (described as x̄ ± s), qualitative data (described as n %), and ranked qualitative data. Student *t*-test, *χ* ^2^ test and Manny-Whitney *U* test were used for comparison of above-mentioned data respectively. Furthermore, the data were analyzed by binary logistic regression (model fitted by means of forward likelihood ratio test) to assess the risk factors of anxiety and depression, taking anxiety and depression as dependent variables, and risk factors as independent variables. The threshold of statistical significance was set as *ɑ* = 0.05.

## Results

**Fig 1:**
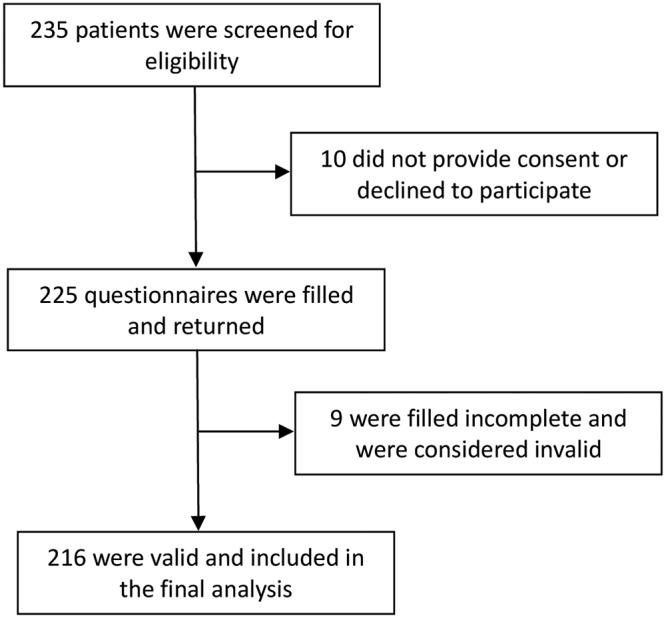
Study flow for enrollment of patients and analysis the valid questionnaires.

Two hundred and thirty-five patients were enrolled in the duration and 225 completed the questionnaires. Finally, 216 questionnaires were considered validated, with a validated response rate of 91.91%. These 216 patients were enrolled for final analysis in the current study (Fig. 1). There were 162 males and 54 females, with an average age of 35.2 ± 10.6 years (range, 15–60 years) (Table 1).

The prevalence of clinical anxiety and clinical depression in this group is 20.4% (44/216) and 21.8% (47/216), respectively. And the prevalence of both clinical anxiety and depression is 15.7% (34/216) (see S1 Dataset).

### Univariate Analysis of Clinical Anxiety and Clinical Depression

Univariate analysis showed that etiology of necrosis, stages of ONFH, Involved femoral head, pain score on VAS and HHS were significant for the dependent variable of clinical anxiety (*P* ≤ 0.05). Employment status, etiology of necrosis, stages of ONFH, gender, comorbid diseases, Involved femoral head and HHS were significant for the dependent variable of clinical depression (*P* < 0.05), as showed in Table 2.

**Table 2:** Univariate analysis of risk factors associated with anxiety/depression in Chinese patients with ONFH.

Independent variables*	Anxiety(n = 216)				Depression(n = 216)			
	No (n,%) = 172	Yes (n,%) = 44	Statistical value	*P*	No (n,%) = 169	Yes (n,%) = 47	Statistical value	*P*
**Employment status**								
Employed	36 (16.7%)	12 (5.6%)	*χ* ^2^ = 7.474	0.058	36 (16.7%)	12 (5.6%)	*χ* ^2^ = 9.908	0.019
On leave	26 (12.0%)	13 (6.0%)			25 (11.6%)	14 (6.5%)		
Unemployed	107 (49.5%)	18 (8.3%)			106 (49.1%)	19 (8.8%)		
Retired	3 (1.4%)	1 (0.5%)			2 (0.9%)	2 (0.9%)		
**Etiology**								
Traumatic	47 (21.8%)	5 (2.3%)	*χ* ^2^ = 8.299	0.040	46 (21.3%)	6 (2.8%)	*χ* ^2^ = 12.887	0.005
Steroid-induced	49 (22.7%)	21 (9.7%)			45 (20.8%)	25 (11.6%)		
Alcohol-induced	35 (16.2%)	10 (4.6%)			36 (16.7%)	9 (4.2%)		
Idiopathic	41 (20.0%)	8 (3.7%)			42 (19.4%)	7 (3.2%)		
**Steinberg Stage**								
II	45 (20.8%)	4 (1.9%)	*Z* = -3.836	0.000	46 (21.3%)	3 (1.4%)	*Z* = -4.679	0.000
III	90 (41.7%)	18 (8.3%)			89 (41.2%)	19 (8.8%)		
IV-V	37 (17.1%)	22 (10.2%)			34 (15.7%)	25 (11.6%)		
**Gender**								
Male	133 (61.6%)	29 (13.4%)	*χ* ^2^ = 2.436	0.119	135 (62.5%)	27 (12.5%)	*χ* ^2^ = 9.871	0.002
Female	39 (18.1%)	15 (6.9%)			34 (15.7%)	20 (9.3%)		
**Comorbid diseases**								
No	113 (52.3%)	23 (10.7%)	*χ* ^2^ = 2.708	0.100	114 (52.8%)	22 (10.2%)	*χ* ^2^ = 6.722	0.010
Yes	59 (27.3%)	21 (9.7%)			55 (25.5%)	25 (11.6%)		
**Involved femoral head**								
Unilateral	100 (46.3%)	12 (5.6%)	*χ* ^2^ = 13.371	0.000	98 (45.4%)	14 (6.5%)	*χ* ^2^ = 11.714	0.001
Bilateral	72 (3.3%)	32 (14.8%)			71 (32.9%)	33 (15.3%)		
Independent variables*	**x̄ ± s**	**x̄ ± s**	*t*	*P*	**x̄ ± s**	**x̄ ± s**	*t*	*P*
**Pain score on VAS**	4.7±1.7	5.3±2.1	-1.971	0.050	4.7±1.7	5.3±1.9	-1.878	0.061
**Harris hip score**	72.6±11.9	65.1±16.4	2.866	0.006	72.5±12.0	65.7±16.0	2.693	0.009

* Only statistically significant independent variables were list in this table

### Multivariate Analysis of Clinical Anxiety and Clinical Depression

Clinical anxiety and Clinical depression were set as dependent variables, while potential risk factors being significant in Univariate analysis were considered as independent variables. Multivariate analyses demonstrated that involved femoral head and stages of ONFH are two independent risk factors associated with clinical anxiety, and then gender, comorbid diseases and stages of ONFH are three independent risk factors associated with clinical depression. The result showed the patients who had bilateral necrosis and advanced stages were more liable to suffer from clinical anxiety; while female, patients who had comorbid diseases and advanced stages were more liable to suffer from clinical depression. OR values concerning all independent variables are demonstrated in Table 3.

**Table 3:** Multivariate logistic regression analysis of anxiety/depression in Chinese patients with ONFH.

*Independent variables*	*β*	*SE*	*Walds*	*P*	*OR* (95% CI)
**Anxiety**					
Involved femoral head	1.153	0.383	9.075	0.003	3.168 (1.496–6.708)
Steinberg Stage			10.928	0.004	
III / II	0.689	0.592	1.355	0.244	1.991(0.624–6.349)
IV-V / II	1.683	0.599	7.895	0.005	5.383(1.664–17.416)
**Depression**					
Gender	1.031	0.402	6.381	0.011	2.853 (1.467–5.778)
Comorbid diseases	1.445	0.399	13.107	0.000	4.243 (1.940–9.278)
Steinberg Stage			23.971	0.000	
III / II	1.174	0.659	3.173	0.075	3.234(0.889–11.769)
IV-V / II	2.831	0.688	16.932	0.000	16.963(4.404–65.331)

## Discussion

ONFH was commonly induced by steroid medication, alcohol abuse and hip injuries. A series of pathological changes might present thereafter, including local insufficient blood supply or ischemia in femoral head, and ischemic necrosis of bone cell, break of bone trabecula, collapse of femoral head and ultimate osteoarthritis [1, 5, 6, 19, 20]. More than 60% patients had bilateral femoral heads involved, which might induce persistent groin pain, restricted motion of the hip joint and abnormal gait posture. Although various treatment protocols have been attempted in past decades, few of them is versatile to relieve the pain, restore the functional joint and delay the need of total hip replacement [21]. The disabling clinical manifestation induced by ONFH, uncertainty of clinical outcome in combination with social, economical and environmental factors, might definitely resulted in patients’ psychological disorders, especially clinical anxiety and clinical depression.

The current study showed higher prevalence of clinical anxiety and clinical depression among the young and middle-aged ONFH Chinese patients with ready for operations, in comparison with that in population (5–10%) [22]. The mental status in Chinese young and middle-aged patients with ONFH should be paid attention to, no matter what kind of Operative procedures (hip preservations or hip arthroplasty) is adopted. Although Etiology of necrosis, Pain scores on VAS, and HHS are associated to clinical anxiety in the univariate analysis, they are merely confounders by controlling other independents. Multivariate analyses demonstrated that involved femoral head and stages of ONFH are two independent risk factors associated with clinical anxiety. Compared with unilateral ONFH, bilateral necrosis had 3.168 times higher risk to get clinical anxiety. Moreover, patients with advanced Steinberg stages (IV-V) are inclined to have anxiety. Furthermore, multivariate analyses indicate that gender, comorbid diseases and advanced stages of ONFH (IV-V) are three independent risk factors associated with clinical depression, and other four independents associated with depression in univariate analysis are confirmed to be confounders. Female patients have 2.853 times higher risks to get clinical depression than the male. The finding is in consistent with the gender tendency of depression in general population [23]. It is known that part of ONFH patients are induced by glucocorticoid medication, due to comorbid systemic lupus erythematosus (SLE), chronic kidney disease or tumor chemotherapy. This study suggested patients with comorbid diseases had a significantly higher risk to get clinical depression than those without comorbid diseases (*OR* = 4.243). Similarly, advanced Steinberg stages of ONFH is risk factor for the presence of clinical depression. This investigation indicated that clinical anxiety and clinical depression are inclined to be intertwined, 15.7% patients of ONFH are suffered from both clinical anxiety and depression according to SAS and SDS scales, the correlation coefficient of Spearman’s rho is 0.68 (p<0.001) for the two psychological disorders (the Spearman’s rho isn’t showed on the results). Due to these high prevalence rate and known factors associated with clinical anxiety and clinical depression, we should pay great attention to the patients’ psychological problems.

Mental disorders not only play negative effects on quality of life and social functions, but also correlate with the occurrence and deterioration of various chronic diseases and unhealthy behaviors. Previous studies displayed that anxiety and depression could cause extensive pain, and have a close relationship with cardiovascular diseases, diabetes mellitus, asthma, smoking and obese [24, 25]. The interaction between physical diseases and psychological impairment has been revealed for long. Clinical anxiety and clinical depression may play a negative role on the long-term effect of the patients who undergo total hip replacement [26, 27]. A large epidemiological survey involving total hip replacement indicated that patients who suffered from anxiety and depression had longer hospital stays, more expensive medical costs, higher abnormal hospital discharge rate and more operation complications [28]. As we known, the difference between clinical anxiety and clinical depression is apparent in terms of their prognosis. Generally, patients with clinical depression are more pronounced tendency to self-harm. Notably, previous study suggested that the prognosis of patients have both clinical anxiety and clinical depression is worse than those patients with clinical depression only [29]. However, in this cross-sectional study, we focused the psychological status preoperatively. It is still unknown whether clinical anxiety and clinical depression could affect the prognosis of treatment for ONFH and whether there is a clear difference between clinical anxiety and clinical depression in patient of osteonecrosis of the femoral head in comparison with other pain or musculoskeletal. calling for further studies. We will investigate the potential damage to prognosis of postoperative functional recovery and effect of psychological intervention in the future.

There are some limitations in this study. One limitation of our study is the prevalence of clinical anxiety and clinical depression in the sampling group may be lower than the actual. The survey bias in this study was inevitable due to the investigation was performed in only one institute. Moreover, a few patients (~8%) refused the investigation or did not fill in the questionnaires as required, leading to no-answer bias. Chinese version SDS standard in this study was set as the most conservative threshold, ≥ 53 scores. When the standard score went down to ≥ 50 scores, the clinical depression rate went up to 26.9% (58/216). Moreover, although we set detail conditions for identify the alcoholic, etiology of necrosis and comorbid diseases at pre-investigation, the actual etiology of necrosis could not be fully reflected at interview; a few respondents even could not define whether themselves administrated with steroid or not previously.

In spite of these limitations, the implications indicated from the results of this investigation study expand the cognition of psychological situation for patients of ONFH and provide an analysis for the potential risk factors lead to clinical anxiety and clinical depression.

## Conclusions

The prevalence of clinical anxiety and clinical depression is quite high in Chinese young and middle-aged patients with ONFH. Patients with bilateral ONFH are inclined to have clinical anxiety, while female patients and patients with comorbid diseases might tend to get clinical depression. Advanced stages of ONFH are independent risk factors for both clinical anxiety and clinical depression.

## Supporting Information

S1 Dataset The de-identified minimal dataset including information for statistical analysis. (SAV)
